# A pathologically diagnosed multinodular and vacuolating neuronal tumor without typical multiple nodules: a case report

**DOI:** 10.3389/fradi.2026.1718759

**Published:** 2026-01-30

**Authors:** Ryota Kitano, Hiroaki Nagashima, Tomoaki Harada, Kazuhiro Tanaka, Keiichiro Uehara, Masamitsu Nishihara, Takashi Sasayama

**Affiliations:** 1 Department of Neurosurgery, Kobe University Graduate School of Medicine, Kobe, Japan; 2 Department of Neurosurgery, Kobe City Nishi-Kobe Medical Center, Kobe, Japan; 3 Department of Diagnostic Pathology, Kobe University Graduate School of Medicine, Kobe, Japan

**Keywords:** 18F-fluciclovine, cortex involvement, HuC/HuD, multinodular and vacuolating neuronal tumors, RAS kinase

## Abstract

Multinodular and vacuolating neuronal tumors (MVNT) are glioneuronal and neuronal tumors classified as grade 1 in the 2021 World Health Organization classification of central nervous system tumors. These tumors are characterized by multiple nodules located between the deep cortex and subcortical white matter. Typically, patients are diagnosed radiologically and do not require treatment. This report describes a rare case of pathologically confirmed MVNT with radiological findings similar to those of low-grade glioma. Moreover, we highlight radiological features useful for differentiating MVNT from low-grade glioma.

## 1 Introduction

Multinodular and vacuolating neuronal tumors (MVNTs) were recently recognized World Health Organization (WHO) grade 1 neuronal tumors and are often discovered incidentally or during work-up for seizures (1). On pathological examination, MVNTs show multiple discrete or coalescent pale nodules involving the deep cortex and subcortical white matter (2). Brain magnetic resonance imaging (MRI) shows multiple hyperintense nodules in the deep cortex and subcortical white matter on T2-weighted and fluid-attenuated inversion recovery images without gadolinium contrast enhancement, mass effect, or surrounding edema (3, 4). These lesions typically remain stable in size during follow-up and usually do not require treatment (4). Therefore, most cases are diagnosed radiologically and rarely confirmed by pathological examination. However, it can be difficult to diagnose MVNT in the absence of distinctive radiological features. We have encountered a pathologically confirmed case that was difficult to differentiate from low-grade glioma (LGG) radiologically. Here we describe this case of MVNT, which mimicked LGG radiologically, and highlight radiological findings that are useful for differentiating MVNT from LGG. This report also reviews the radiologic features of MVNT without multiple nodules based on previously reported pathologically confirmed cases.

## 2 Case presentation

**Figure 1:**
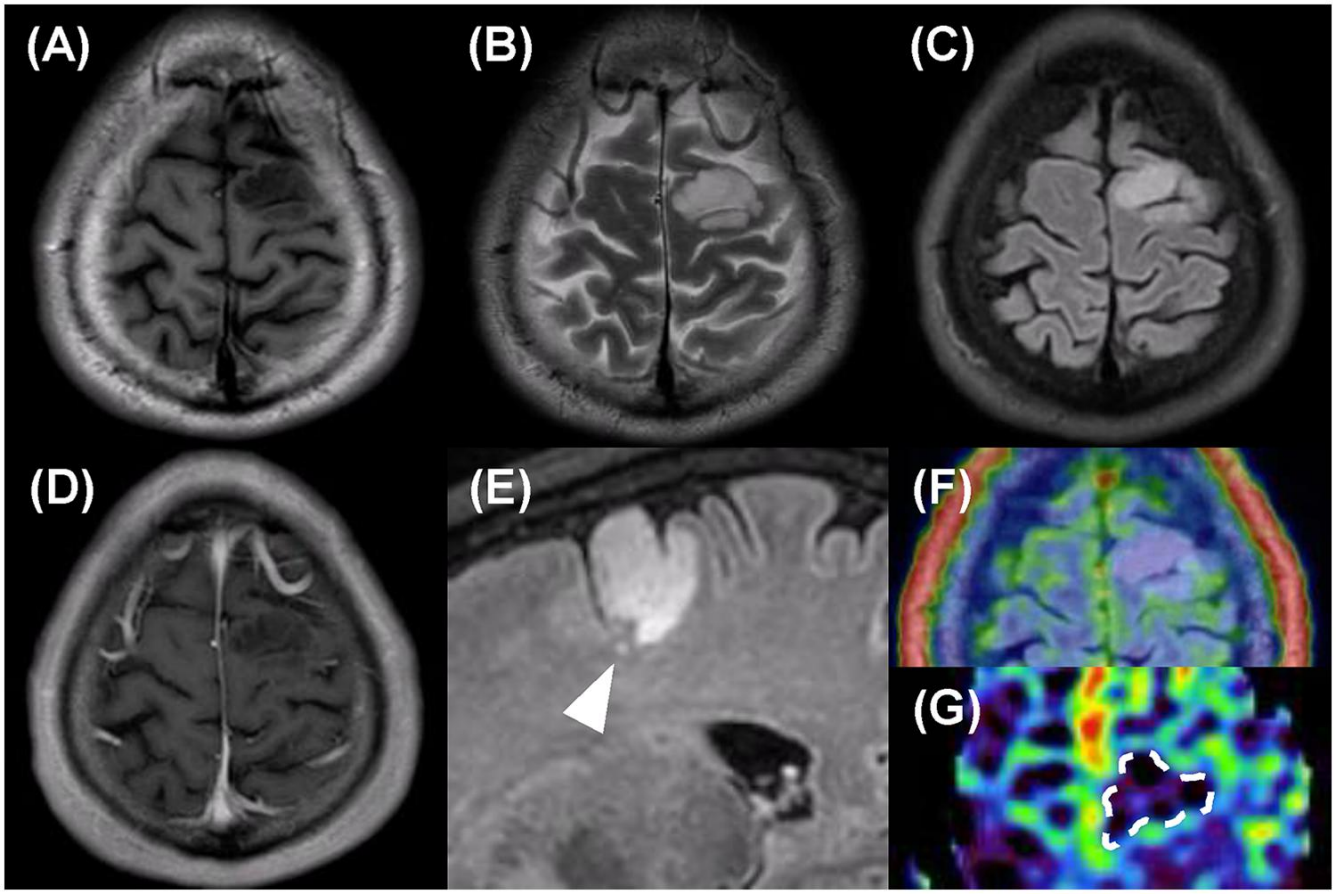
Findings on initial brain magnetic resonance images. **(A)** Axial T1-weighted image showing heterogeneous hypointensity in the precentral gyrus. **(B)** Axial T2-weighted image showing heterogeneous hyperintensity. **(C)** Axial FLAIR image with the same findings as those on the T2-weighted image. **(D)** Axial T1-weighted image with contrast showing no enhancement. **(E)** A sagittal thin-slice FLAIR image showing the hyperintense lesion and one hyperintense nodule (arrowhead) in the white matter without mass effect. **(F)** ^18^F-fluciclovine positron emission scan showing focal hypometabolism. **(G)** Arterial spin labeling showed decreased intratumoral blood flow (white dashed line).

**Figure 2:**
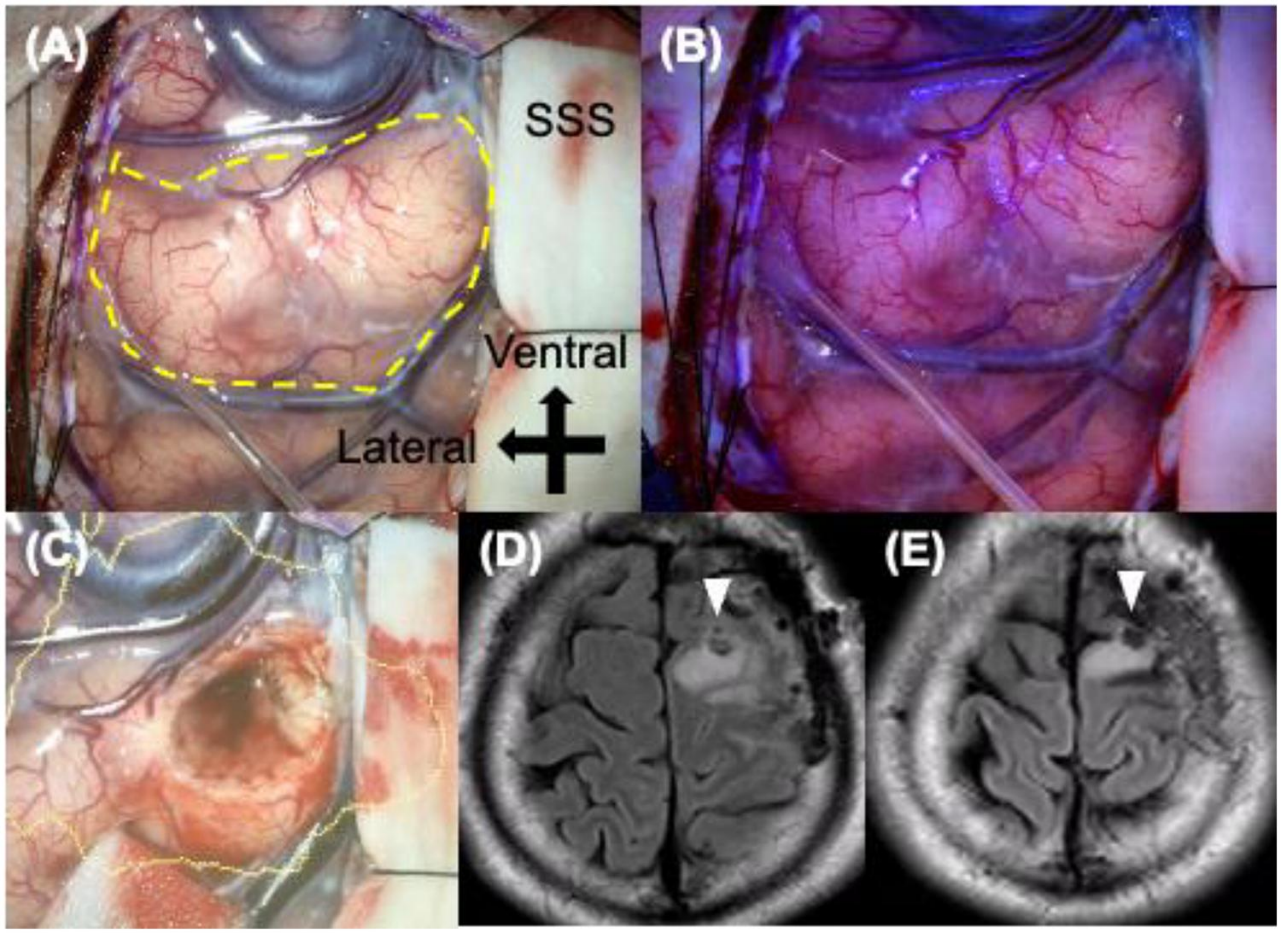
Intraoperative view and postoperative images. **(A)** The precentral gyrus is located in the center of the image and appears whitish with normal cortex (yellow dashed line). **(B)** The gyrus does not show fluorescence with 5-aminolevulinic acid. **(C)** The ventral area of the precentral gyrus was resected. **(D)** A postoperative FLAIR image showing the resected cavity in the same region as observed intraoperatively (white arrow). **(E)** Six-month follow-up FLAIR showed no progression (white arrow). SSS, superior sagittal sinus.

**Figure 3:**
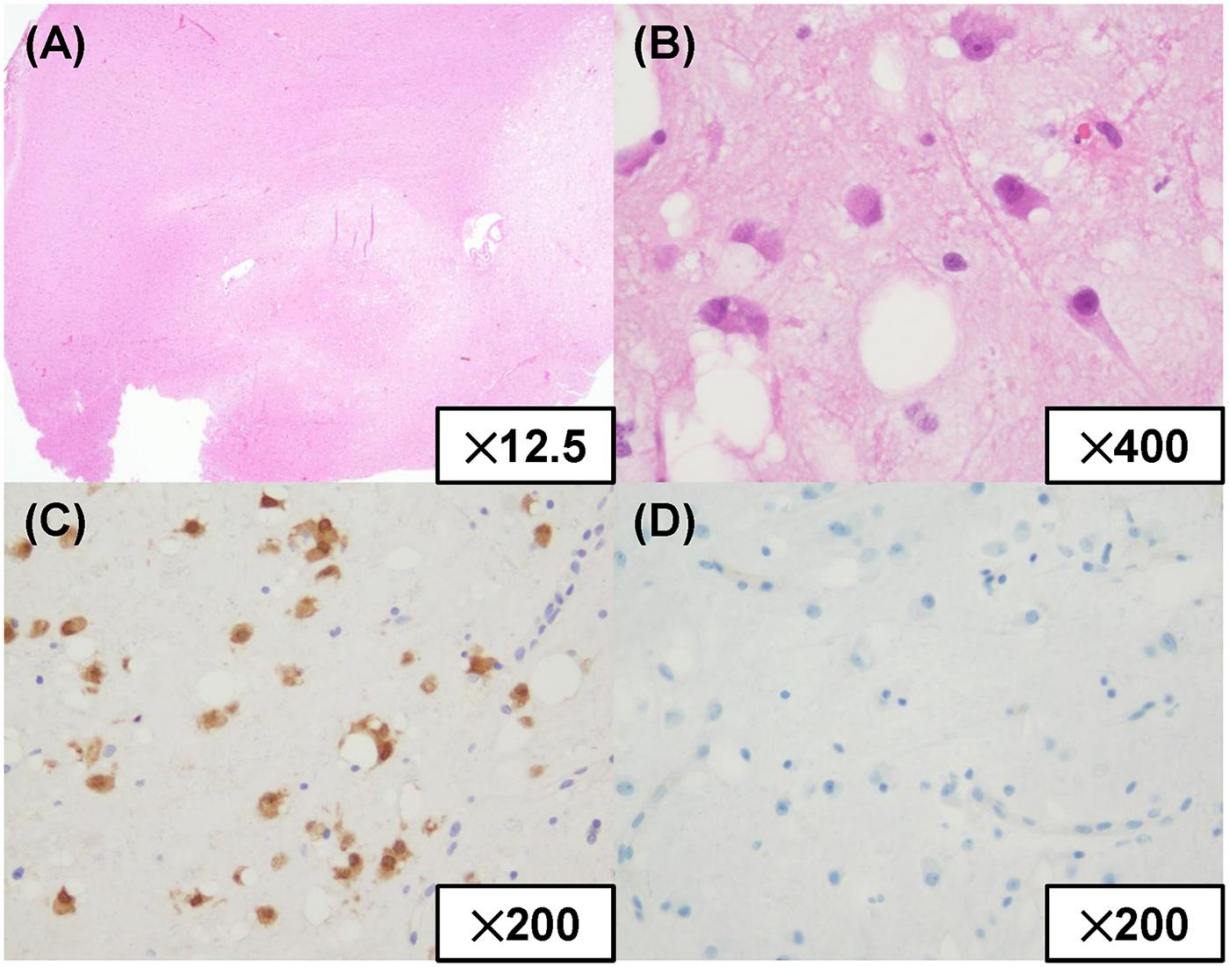
Histopathological images. **(A)** Low-power view of a hematoxylin–eosin stained section showing a normal cortex and multiple pale nodules in the subcortical layer. **(B)** High-power view of a hematoxylin–eosin stained section showing cytoplasmic and pericellular vacuolating changes. **(C)** Tumor cells expressing HuC/HuD. **(D)** The nodules were negative for Ki-67 immunostaining.

The patient was a man in his 30s who presented with occipital headache for a long period. His past medical history included cough-variant asthma and allergic rhinitis and he has antihistamines, inhaled corticosteroids and nonsteroidal anti-inflammatory drugs. A screening computed tomography scan of the brain had demonstrated a low-density lesion in the left precentral gyrus, and he was referred to our hospital. Levetiracetam had been started by the previous doctor for seizure prophylaxis. Neurological examination and blood tests were normal. Brain MRI was performed using a 3.0-T Philips Ingenia system (Philips Healthcare, Best, the Netherlands) equipped with a 15-channel SENSE head coil. The MRI protocol consisted of conventional sequences, including T1-weighted imaging (T1WI), T2-weighted imaging (T2WI), fluid-attenuated inversion recovery (FLAIR), diffusion-weighted imaging (DWI) with apparent diffusion coefficient (ADC) maps and contrast-enhanced T1WI. The MRI protocol consisted of: T1-weighted SE: TR = 521 ms, TE = 8 ms, flip angle = 68°, T2-weighted TRA SE: TR = 4,022 ms, TE = 102 ms, flip angle = 90°, T1-weighted 3D Gd GR: TR = 5 ms, TE = 2 ms, flip angle = 15°, FLAIR: TR = 4,800 ms, TE = 340 ms, TI = 1,650 ms, flip angle = 90°, DWI (EPI): b = 0/1,000 s/mm^2^, TR = 3,500 ms, TE = 75 ms, flip angle = 90°. The slice thickness was 4–5 mm for all sequences except for contrast-enhanced T1WI, which was acquired with a slice thickness of 0.9 mm. Thin-slice FLAIR was acquired using 3D BrainView FLAIR sequence with 1.1-mm isotropic voxels. Axial images demonstrated an area in the left precentral gyrus that was heterogeneously hypointense on T1WI and hyperintense on T2WI (Figures 1A,B). An axial FLAIR image showed the same hyperintense area, and the nodules were unremarkable (Figure 1C). DWI demonstrated isointensity to the cortex, that the area was not enhanced, and that there was no mass effect or surrounding edema (Figure 1D). Therefore, the area was initially interpreted as an LGG. Thin-slice sagittal FLAIR imaging, performed retrospectively after the final diagnosis, revealed a very small hyperintense nodule under a homogeneous hyperintense area (Figure 1E). Arterial spin labeling showed that intratumoral blood flow was decreased (Figure 1F). ^18^F-Fluciclovine positron emission tomography (PET)/MRI revealed hypometabolism in the lesion (Figure 1G). We performed an open surgical biopsy with craniotomy. Intraoperatively, the lesion appeared to be normal cortical matter with whitish subcortical white matter and was negative for 5-aminolevulinic acid fluorescence (Figures 2A,B). The anteroventral portion of the lesion was resected for pathological examination (Figure 2C). The resected lesion was also examined by MRI (Figure 2D). A low-power view of hematoxylin–eosin-stained sections showed normal overlying cortex and multiple pale nodules in the subcortical layer (Figure 3A). A high-power view with hematoxylin–eosin staining demonstrated large round nuclei with cytoplasmic and pericellular vacuolization (Figure 3B). Immunohistochemical staining showed positivity for HuC/HuD and negativity for glial fibrillary acidic protein and Ki-67 (Figures 3C,D). Based on the histomorphology and immunohistochemistry results, the patient was diagnosed with MVNT. No progression of the lesion was observed over 12 months postoperatively, and no further treatment was deemed necessary.

## 3 Discussion

We encountered a rare case of MVNT without typical radiological evidence of multiple nodules that was diagnosed based on pathological findings. This case highlights the diagnostic challenge of MVNT, the radiological findings of which can mimic diffuse LGG. Histopathology remains the gold standard for definitive diagnosis. Careful evaluation with MRI, particularly thin-slice FLAIR imaging, can reveal the microcystic nodules characteristic of MVNT, thereby avoiding misdiagnosis. Furthermore, sagittal and coronal views may offer valuable insights that complement findings on axial thin-slice T2-weighted and FLAIR images.

MVNT was first described in 2013 by Huse et al. in their report of a case series of patients who presented with a focal or complex seizure (2). Thereafter, several additional case reports were published, leading to inclusion of MVNT in the 2016 WHO classification of central nervous system tumors (5–7), where it was categorized as a pattern of ganglion cell tumors. However, it is unknown whether the tumor represents a neoplastic or malformation process (4). MVNTs are recognized as clonal neoplasms in the MAPK pathway with mutations in MAPK2K1 and BRAF (excluding V600E) and FGFR2 fusions (8). This tumor affects young adults (with a reported mean age of 32.6 ± 17.6 years) and has a slight male predominance (1.3:1) (4).

MVNT is characterized by cytoplasmic and pericellular vacuolization, shows no mitotic activity, and is located between the deep cortex and subcortical white matter. The tumor is immunochemically positive for HuC/HuD, a neuron-restricted marker that plays a role in neuronal differentiation and maintenance (2). On brain MRI, MVNT demonstrates characteristic features, including multiple clusters of discrete or coalescent nodules between the deep cortex and subcortical white matter that are hyperintense on T2WI and FLAIR without mass effect, surrounding edema, or contrast enhancement, reflecting its pathological features (3). In view of these benign features, MVNT is often managed with radiological follow-up (4), and cases that require biopsy because of difficulty with radiological diagnosis are rare (3).

To date, 41 cases of pathologically confirmed MVNT have been reported (2, 5–7, 9–21). Most of these MVNTs were located in the temporal lobe (34.2%), followed by the parietal lobe (24.4%), frontal lobe (14.6%), and occipital lobe (12.2%). MVNTs may also present as infratentorial lesions (14.6%) (4). The most common symptom being seizure (56.1%), followed by headache (9.8%) and dizziness (9.8%). No symptoms were observed in 4.9% of cases. Furthermore, a large case series (3) has been reported in which 23.4% of patients with radiologically diagnosed MVNT were asymptomatic. Given that asymptomatic patients do not require surgical treatment, the ratio of asymptomatic patients could be lower in pathological confirmed cases than in radiologically diagnosed cases.

Few or multiple nodules were seen in most cases, and solid lesions were also observed (95.8%, 23/24 cases. Solid lesions were located in the subcortical and subcortical white matter, and radiological imaging often showed a partially preserved overlying cortex (62.5%, 15/23 cases). T2-weighted imaging is more suitable than FLAIR for evaluating the cortex (6). The cortex was more preserved for lesions located in the cerebral hemispheres than in the medial temporal lobe, including the hippocampus, amygdala, and parahippocampal gyrus (92.9% vs. 25%) (2, 5–7, 9–21). Only five cases without nodules have been reported (6, 12, 17). Solid-appearing lesions may mimic LGG, for which a different treatment strategy is required. In some cases, the lesion was resected with a preoperative diagnosis of LGG, but histopathology revealed MVNT (15, 18). Table 1 summarizes the published reports of cases that were similar to LGG radiologically but were confirmed histopathologically to be MVNT (2, 5, 6, 9, 10, 12, 13, 15, 17, 18). There have been reports of two cases in the medial temporal lobe (12, 17) and reports of three cases in a cerebral hemisphere (6, 12). All three cases located in a cerebral hemisphere demonstrated a partially preserved cortex. Therefore, a preserved cortex may aid in differentiating between MVNT and LGG.

**Table 1:** Clinical and demographic characteristics.

Patient No.	Age	Sex	Location	Symptoms	MRI finding			Prognosis	Author
					Multiple nodules	Solid lesion	Cortical involvement		
1	38	M	Medial temporal	Dizziness, loss of attention	Yes	Yes	NA	No progression	Huse et al. (2)
2	64	M	Temporal	Staring and mumbling	No	NA	NA	No progression	Huse et al. (2)
3	71	F	Temporal	Dysarthria	Yes	Yes	No	No progression	Bodi et al. (5)
4	37	M	Parietal	Seizure	No	Yes	No	No progression	Fukushima et al. (6)
5	41	M	Medial temporal	Seizure	Yes	Yes	Yes	NA	Yamaguchi et al. (9)
6	53	M	Medial temporal	Epilepsy	Yes	Yes	No	NA	Nunes et al. (10)
7	67	F	Parietal	Seizure with visual symptoms	No	Yes	No	No progression	Thom et al. (12)
8	48	F	Temporal	Seizure with aphasia	No	Yes	No	No progression	Thom et al. (12)
9	48	NA	Medial temporal	Seizure	Yes	Yes	Yes	No progression	Bascarevic et al. (13)
10	58	F	Temporal	Headache	Yes	Yes	No	No progression	Alizada et al. (15)
11	5	M	Basal ganglia	Cerebral palsy	No	Yes	Yes	NA	Turner et al. (17)
12	45	F	Parietal	Seizure	Yes	Yes	No	No progression	Bagatto et al. (18)
13	30s	M	Frontal	Headache	No	Yes	No	No progression	Present case

NA, not available.

In our patient, the lesion was located in the precentral gyrus, and FLAIR demonstrated a solid lesion with a preserved overlying cortex. The outermost layer of the cortex, which appeared to be involved on FLAIR imaging, was preserved histopathologically. T2-weighted images confirmed a preserved cortex. Small nodules were confirmed pathologically and corresponded to the radiological findings. We resected the solid lesion detected on FLAIR. Histopathology showed multiple nodules in the deep cortex and subcortical white matter without edema. The histopathological multiple nodules did not explain the radiological solid lesion. Our specimen included nodules that were approximately 2.5 mm in diameter. The lesion was too small to be detected by MRI, and the cluster of nodules was demonstrated as a solid lesion. Further studies are needed to resolve this inconsistency. Indeed, in the present case, reviewing the thin-slice FLAIR images after the pathological diagnosis revealed one tiny nodule (Figure 1E). Therefore, thin-slice FLAIR might be helpful for the preoperative diagnosis.

Several reports have described the use of advanced multiparametric MRI and PET to differentiate MVNT. The case series mentioned earlier described use of advanced MRI and reported the mean apparent diffusion coefficient, cerebral blood flow on arterial spin labeling, and the choline to N-acetyl aspartate ratio on magnetic resonance spectroscopy to be 1.13, 1.01, and 0.7, respectively (3). In our case, cerebral blood flow was decreased in comparison with the contralateral side. There have been reports of two pathologically confirmed cases that underwent ^18^F-fluorodeoxyglucose PET (10, 13) and one of a case that was investigated by ^11^C-methionine PET (6). Hypometabolism was demonstrated in both the cases in which ^18^F-fluorodeoxyglucose PET was performed. In contrast, ^11^C-methionine PET did not show an increase in uptake. In our case, there was limited uptake of ^18^F-fluciclovine on PET (22). Multimodal imaging may be helpful for preoperative diagnosis of MVNT without typical MRI findings.

MVNT generally has a favorable prognosis and often requires no treatment, as it can usually be diagnosed radiologically based on its characteristic imaging features. Typical MVNT presents as multiple clusters of discrete or coalescent nodules located between the deep cortex and subcortical white matter, appearing hyperintense on T2WI and FLAIR without mass effect, edema, or contrast enhancement (4). In contrast, LGG involves both cortical and subcortical structures pathologically and radiologically, requires surgical and adjuvant treatments, and typically manifests as a homogeneous T2/FLAIR hyperintense lesion without enhancement (23, 24). Importantly, LGG lacks the multinodular pattern characteristic of MVNT. However, MVNT cases without obvious nodular clusters may resemble LGG, leading to diagnostic difficulty. In the present study, we reported an MVNT case that mimicked LGG and reviewed previously documented pathologically confirmed cases with similar radiological features. MVNT may present as a solid-appearing lesion with only a few tiny nodules, and thin-slice FLAIR can improve the sensitivity for detecting these subtle components, allowing for more accurate diagnosis. A key differentiating feature is that MVNT preserves the overlying cortex, whereas LGG typically infiltrates the cortical ribbon. Our review confirmed that cortical preservation over solid lesions was frequently observed in MVNT cases resembling LGG, supporting its value as a practical radiological discriminator.

## 4 Conclusion

We reported an atypical radiological case of MVNT and summarized the radiological features of histopathologically proven MVNT. Multiple nodules and solid lesions are typical of MVNT, and nodules can be missed on imaging. MVNT should be considered in the differential diagnosis of lesions suggestive of glioma. Careful inspection of thin-slice FLAIR images for microcystic nodules is essential for identifying MVNT and guiding appropriate management.

## Data Availability

The raw data supporting the conclusions of this article will be made available by the authors, without undue reservation.
